# Association of medical conditions and firearm suicide among legal handgun purchasers in California: a case–control study

**DOI:** 10.1186/s40621-023-00437-6

**Published:** 2023-06-16

**Authors:** Julia P. Schleimer, Rose M. C. Kagawa, Hannah S. Laqueur

**Affiliations:** 1 Violence Prevention Research Program, University of California, Davis, 2315 Stockton Blvd., Sacramento, CA 95817 USA; 2 California Firearm Violence Research Center, Sacramento, CA USA

**Keywords:** Firearms, Suicide, Suicide prevention, Mental health, Selection bias

## Abstract

**Background:**

Suicide is a pressing public health problem, and firearm owners are at especially elevated risk. Certain health conditions are markers of suicide risk, but more research is needed on clinical risk markers for suicide among firearm owners specifically. Our goal was to examine associations of emergency department and inpatient hospital visits for behavioral and physical health conditions with firearm suicide among handgun purchasers.

**Methods:**

This was a case–control study of 5415 legal handgun purchasers in California who died between January 1, 2008, and December 31, 2013. Cases were firearm suicide decedents; controls were motor vehicle crash decedents. Exposures were emergency department and hospital visits for six categories of health diagnoses in the 3 years prior to death. To account for selection bias due to deceased controls, we used probabilistic quantitative bias analysis to generate bias-adjusted estimates.

**Results:**

There were 3862 firearm suicide decedents and 1553 motor vehicle crash decedents. In multivariable models, suicidal ideation/attempt (OR 4.92; 95% CI 3.27–7.40), mental illness (OR 1.97; 95% CI 1.60–2.43), drug use disorder (OR 1.40; 95% CI 1.05–1.88), pain (OR 1.34; 95% CI 1.07–1.69), and alcohol use disorder (OR 1.29; 95% CI 1.01–1.65) were associated with higher odds of firearm suicide. When adjusting for all conditions simultaneously, only the associations for suicidal ideation/attempt and mental illness remained significant. Quantitative bias analysis indicated that observed associations were generally biased downward. For example, the bias-adjusted OR for suicidal ideation/attempt was 8.39 (95% simulation interval 5.46–13.04), almost twice that of the observed OR.

**Conclusions:**

Diagnoses for behavioral health conditions were markers for firearm suicide risk among handgun purchasers, even for conservative estimates that did not adjust for selection bias. Encounters with the healthcare system may provide opportunities to identify firearm owners at high risk of suicide.

**Supplementary Information:**

The online version contains supplementary material available at 10.1186/s40621-023-00437-6.

## Background

Suicide is a leading cause of death in the USA. Nationally, suicide rates increased by 30% from 2000 (10.4 per 100,000) to 2020 (13.5 per 100,000) (Garnett et al. 2022), when suicide resulted in 45,979 deaths (CDC WISQARS 2022). Firearms are the most commonly used and most lethal method of suicide, accounting for less than 5% of suicide acts (non-fatal and fatal suicide attempts) but over 50% of all suicide deaths (Conner et al. 2019a). Almost 90% of suicide attempts with a firearm result in death (Conner et al. 2019a), a case fatality well above the next most lethal methods (drowning, 56% and hanging, 53%) (Conner et al. 2019a).

Firearm access is a well-documented risk factor for firearm suicide. Case–control and cohort studies alike have consistently demonstrated that those with personal or household firearm access are substantially more likely to die by suicide compared with those without firearm access (Wintemute et al. 1999; Kellermann et al. 1992; Anglemyer et al. 2014). The excess risk of suicide among those with access to firearms has been primarily attributed to a higher risk of firearm suicide (Wintemute et al. 1999; Kellermann et al. 1992; Studdert et al. 2020).

Strategies to reduce suicide deaths include limiting access to firearms. Due to the legal and cultural milieu in the USA, these strategies are often risk-based, rather than universal. For example, current guidelines for health professionals recommend firearm safety counseling for patients with behavioral, demographic, or clinical risk markers (Pallin et al. 2019; Pallin and Barnhorst 2021). While prior research suggests that health professionals are uniquely positioned to identify and counsel patients at increased risk of suicide, we know relatively little about clinical risk factors for suicide among firearm owners as a group.

Prior research among the general population and certain patient populations (e.g., clinical samples, Medicaid beneficiaries) has found that many individuals who died by suicide had a recent healthcare visit prior to death and that certain health conditions are salient markers of suicide risk (Hempstead et al. 2013; Ahmedani et al. 2014). Behavioral health diagnoses, including depression, anxiety, and substance use disorders, are strongly associated with suicide, including firearm suicide (Sivaraman et al. 2022; Ilgen et al. 2010; Bohnert et al. 2017; Olfson et al. 2021; Too et al. 2019). Physical health conditions too, such as pain and chronic illnesses, have been associated with increased risk of suicide (Hempstead et al. 2013; Goldman-Mellor et al. 2021; Ilgen et al. 2013; Ahmedani et al. 2017), highlighting the potential for healthcare encounters to prevent suicide among individuals who might not otherwise be identified as being at elevated risk due to a mental illness.

However, prior research among the general population or specific patient populations may not generalize to firearm owners because of differences in the underlying population characteristics (e.g., demographics), distribution of effect modifiers, etiology of suicide risk, or method of suicide most commonly used. For example, firearm owners are more often male, White, and married than the general population and more likely to live in rural areas (Miller et al. 2022; Mitchell 2017). These characteristics may be related to access to and engagement with healthcare, norms about help seeking, and risk of suicide (Hempstead et al. 2013; Douthit et al. 2015). Further, firearm owners are substantially more likely to use firearms as a method of suicide than are non-firearm owners (Studdert et al. 2020). Because firearms are highly lethal, this may result in a relatively shorter window of opportunity for individuals at high risk of firearm suicide to be diagnosed with health conditions or connect with healthcare professionals (Sivaraman et al. 2022). Prior research has found that clinical conditions are less strongly associated with firearm suicide than with non-firearm suicide (Boggs et al. 2018). It is therefore important to identify whether and what clinical conditions are associated with risk for firearm suicide among firearm owners as a group.

We conducted a descriptive case–control study of legal handgun purchasers in California to examine the associations of emergency department (ED) and inpatient hospital visits (hereafter collectively referred to as “hospital visits”) for behavioral and physical health conditions with firearm suicide. This is the first study, to our knowledge, to extend prior research on clinical risk factors for suicide to handgun purchasers, a group at especially high risk of suicide. Results will contribute to the evidence on risk markers for firearm suicide and inform future etiologic investigations.

## Methods

### Setting and population

The study population for this case–control study was legal handgun purchasers in California who died between January 1, 2008, and December 31, 2013. The sample was first drawn from legal handgun purchasers in California between January 1, 1996, and December 31, 2013, identified via California’s Dealer’s Record of Sale (DROS) database. DROS records are maintained by the California Department of Justice (CA DOJ) and include all legal handgun purchases in the state. Record-keeping for long gun sales was not required until 2014; long gun-only purchasers were therefore excluded from the present study. As in prior research (Kagawa et al. 2020; Schleimer et al. 2021), we linked handgun purchasers to the California Department of Public Health’s Death Statistical Master File, matching probabilistically on name, date of birth, and sex, to identify the subset of purchasers who died between 2008 and 2013. The California Office of Statewide Planning and Development (OSHPD) then further linked these decedents to statewide ED and hospital discharge data, returning all such visits for each decedent within the three years prior to death. The legal age of handgun purchase is 21 years; we therefore excluded a small number of individuals under age 21 at the time of death (*n* = 13). We were limited to the years 2008 to 2013 because OSHPD did not make multi-year linked death data available before 2005 or after 2013; we further limited the time period to 2008 so that a full three years of hospital visit data could be available for all cases and controls. Our resulting study population included legal handgun purchasers aged 21 years and older who died between 2008 and 2013 along with all of their corresponding hospital visits, if any, in the three years prior to death.

### Selection of cases and control

Cases were defined as all those who died by firearm suicide during the study period (International Classification of Diseases, Tenth Revision codes X72–X74, consistent with prior research research) (Goldman-Mellor et al. 2021). Controls were all those who died from motor vehicle crashes (MVC) as an occupant or driver (International Classification of Diseases [ICD], Tenth Revision codes V02–V04; V09.0; V09.2; V12–V14; V19.0–V19.2; V19.4–V19.6; V20–V79; V80.3–V80.5; V81.0–V81.1; V82.0–V82.1; V83–V86; V87.0–V87.8; V88.0–V88.8; V89.0; V89.2, consistent with prior research) (Cunningham et al. 2018). “Motor vehicle” is defined as a car, bus, truck, motorcycle, or other transport vehicle. We excluded MVC deaths due to intentional self-harm, assault, or undetermined intent. We included all eligible cases and controls in our study. Cases and controls were not matched.

We selected this control group to minimize biases resulting from the use of deceased controls, as we did not have data on ED and hospital visits among non-deceased handgun purchasers. Deceased controls are not representative of the source population that gave rise to the cases (the general population of legal handgun purchasers in this case) (Wacholder et al. 1992). This selection bias will be exacerbated if the cause of death for the control group is associated with the exposures under study (Rothman et al. 2008). While no group of decedents is perfectly representative of the source population, MVC decedents may be more representative of the source population than individuals who died of other causes as MVC deaths are likely more unexpected than deaths from other causes, especially chronic diseases (e.g., cancer) in which patients often experience a protracted period of decline, perhaps with frequent hospital visits. We examined the degree of bias in our control group by comparing them to handgun purchasers who died of other causes and to the general (living) California population of handgun and non-handgun owners (as it was not possible to identify handgun ownership status among non-deceased individuals with hospital visits). We additionally generated bias-adjusted estimates to account for non-representativeness of the control group by conducting a summary-level probabilistic quantitative selection bias analysis (described in detail below) (Tordoff et al. 2019; Banack et al. 2022; Lash et al. 2009).

### Exposures

We examined a number of health diagnoses that have been associated with suicide or firearm suicide in the prior literature (Hempstead et al. 2013; Sivaraman et al. 2022; Ilgen et al. 2010, 2013; Bohnert et al. 2017; Olfson et al. 2021; Goldman-Mellor et al. 2021, 2019). These included six categories: (1) mental illnesses (depression, anxiety, post-traumatic stress disorder [PTSD], bipolar disorder, and schizophrenia); (2) substance use (alcohol use disorder/poisoning, drug use disorder/poisoning, and a subset of drug use disorders/poisonings involving opioids, stimulants, cannabis, or sedatives); (3) pain (rheumatoid arthritis, osteoarthritis; migraine, chronic headache; fibromyalgia, chronic pain, fatigue); (4) chronic disease (acute myocardial infarction [MI]; heart failure; hypertension; stroke, transient ischemic attack; asthma; chronic obstructive pulmonary disorder [COPD]; diabetes; traumatic brain injury [TBI]; epilepsy; cancer [breast, colorectal, endometrial, lung, and prostate]); (5) assault; and (6) suicidal ideation and suicidal attempt. We limited drug poisonings to those that were unintentional. We identified hospital visits for these conditions using ICD-9-Clinical Modification (CM) diagnosis codes and external cause of injury codes in any diagnostic field. Diagnostic codes were identified from published sources (Heslin et al. 2015; CDC’s Drug Overdose Surveillance and Epidemiology (DOSE) System 2022; National Center for Injury Prevention and Control 2013; Centers for Medicare and Medicaid Services 2022; Oliva et al. 2017; Fingar et al. 2018). Specific ICD-9-CM codes for each condition are in eTable 1, Additional file 1.

### Analysis

We estimated the odds that a handgun purchaser was a case (firearm suicide decedent) relative to a control (MVC decedent) using logistic regression with robust standard errors. To adjust for demographic and temporal differences between cases and controls, we included the following individual-level covariates in multivariable models: sex (male or female), age at death in years (continuous), calendar year of death (continuous), marital status (married/domestic partnership; divorced; never married; widowed; unknown), and educational attainment (high school degree or less; some college; bachelor’s degree; associate’s degree; graduate degree; unknown). We also adjusted for urbanicity of decedents’ county of residence using the US Department of Agriculture’s 2003 Rural–Urban Continuum Codes (counties in a metropolitan area with 1 million population or more [“large metro”]; counties in metro area of fewer than 1 million population [“small metro”]; and “non-metro” counties) (United States Department of Agriculture 2021). Covariate data originated from mortality records. We excluded people with missing sex or county of residence (excluding 0.9% of observations in total). We first estimated separate models for each of the six clinical categories, adjusting for the covariates listed above. We then estimated a model that included all six clinical categories together, along with the same set of covariates. While our goal was descriptive, we nonetheless included covariates to more accurately parse the associations under study and explore which specific diagnoses were driving observed associations. To further understand the relationship between these clinical categories and firearm suicide, we estimated models that disaggregated the components of each subcategory, including each component in separate models and then in models that simultaneously adjusted for the other components of that category. For example, for the mental illness category, we examined the associations of depression, anxiety, PTSD, bipolar disorder, and schizophrenia in separate models and in one model with all variables entered simultaneously.

We additionally conducted a quantitative bias analysis (QBA) to generate bias-adjusted estimates that account for selection bias introduced through our use of deceased controls (Banack et al. 2022; Lash et al. 2009). We estimated the bias-adjusted OR for the association between each health condition and firearm suicide as ${\widehat{\text{OR}}}_{\text{total error}}=\text{exp}\left(\text{ln}({\widehat{\text{OR}}}_{\text{adj}})+N\left(\text{0,1}\right)\times \text{SE}(\text{ln}({\widehat{OR}}_{\text{observed}})\right)$ where ${\widehat{\text{OR}}}_{\text{adj}}= {\widehat{\text{OR}}}_{\text{observed}}\times {\text{OR}}_{\text{select}}$; ${\widehat{\text{OR}}}_{\text{observed}}$ is the observed OR (from regression models adjusted for sex, age, death year, marital status, educational attainment, urbanicity); and ${\text{OR}}_{\text{select}}=\frac{{S}_{\text{case},0}{S}_{\text{control},1}}{{S}_{\text{case},1}{S}_{\text{control},0}}$ where *S* is a selection proportion and exposed and unexposed are indexed by 1 and 0, respectively (Tordoff et al. 2019; Banack et al. 2022; Lash et al. 2009). The ${\widehat{\text{OR}}}_{\text{total error}}$ accounts for both systematic bias and random error, the latter through the term: $N\left(\text{0,1}\right)\times \text{SE}(\text{ln}({\widehat{\text{OR}}}_{\text{observed}})$ where $N\left(\text{0,1}\right)$ is a standard normal distribution. We assumed all cases were selected, thus ${\text{OR}}_{\text{select}}$ simplified to $\frac{{S}_{\text{control},1}}{{S}_{\text{control},0}}$. In the absence of known selection probabilities for the control group, we used the abovementioned comparison of exposure rates among our control group (MVC decedents) and the general living California population as a benchmark for ${\text{OR}}_{\text{select}}$, i.e., using the ratio of exposure rates among MVC decedents to exposure rates among the general living California population. When controls with exposure were over-represented in our sample, ${\text{OR}}_{\text{select}}>1$ and ${\widehat{\text{OR}}}_{\text{adj}}$ was adjusted upward. When controls with exposure were under-represented in our sample, ${\text{OR}}_{\text{select}}<1$ and ${\widehat{\text{OR}}}_{\text{adj}}$ was adjusted downward. We used a Monte Carlo simulation with 50,000 iterations, specifying a triangular distribution with mode equal to ${\text{OR}}_{\text{select}}$ and lower and upper limits ranging from 20% lower to 20% higher than the mode to reflect uncertainty in true selection probabilities. The median of this distribution is ${\widehat{\text{OR}}}_{\text{total error}}$, and the 2.5th and 97.5th percentiles constitute the 95% simulation interval. Example R code is provided in Additional file 1.

All analyses were conducted in R version 4.0.0 (R Foundation for Statistical Computing, Vienna, Austria). The University of California, Davis Institutional Review Board approved this study.

## Results

Our study population included 3862 firearm suicide decedents (representing 77% of all suicide decedents) and 1553 MVC decedents (Table 1). A large majority of both groups were male. Firearm suicide decedents had higher educational attainment on average, were more often divorced or widowed compared with controls, and were more likely to live in a large metro area. Almost half of firearm suicide decedents (45.8%) and MVC decedents (48.9%) had any hospital visit in the three years prior to death. Compared with controls, those who died by firearm suicide were more likely to have a hospital visit for suicidal ideation or attempt, mental illness, most types of substance use, and pain (Table 1). The most common visits among firearm suicide decedents were for chronic diseases (25.1%), mental illness (15.2%), and substance use (10.9%), followed by pain (9.9%) and suicidal ideation/attempt (7.5%). One in five had a visit for either suicidal ideation/attempt, mental illness, or substance use (20.0%). Results suggest that handgun purchasers who died in motor vehicle crashes were likely more representative of the proxied source population than were handgun purchasers who died of other causes (eTable 2, Additional file 1).

**Table 1:** Description of legal handgun purchasers who died by firearm suicide or MVC, 1/1/2008–12/31/2013

	Firearm suicide, *N* = 3862	Motor vehicle crash, *N* = 1553
	No. (%)	No. (%)
*Sociodemographic characteristics*		
Age at death in years, mean (SD)	56.0 (16.7)	50.8 (15.8)
Sex		
Female	316 (8.2%)	132 (8.5%)
Male	3546 (91.8%)	1421 (91.5%)
Education status		
High school or less	1445 (37.4%)	760 (48.9%)
Some college	941 (24.4%)	401 (25.8%)
Associates	283 (7.3%)	125 (8.0%)
Bachelor	703 (18.2%)	155 (10.0%)
Graduate	408 (10.6%)	90 (5.8%)
Unknown	82 (2.1%)	22 (1.4%)
Marital status		
Divorced	960 (24.9%)	307 (19.8%)
Married/partner	1580 (40.9%)	771 (49.6%)
Never married	963 (24.9%)	383 (24.7%)
Widowed	306 (7.9%)	82 (5.3%)
Unknown	53 (1.4%)	10 (0.6%)
Rural–urban status		
Large metro	2735 (70.8%)	962 (61.9%)
Small metro	894 (23.1%)	469 (30.2%)
Rural	233 (6.0%)	122 (7.9%)
*Health conditions*^a^		
Any hospital visit	1770 (45.8%)	760 (48.9%)
Suicidal ideation/attempt	291 (7.5%)	27 (1.7%)
Suicidal ideation	86 (2.2%)	16 (1.0%)
Suicide attempt	223 (5.8%)	15 (1.0%)
Mental illness	588 (15.2%)	124 (8.0%)
Depression	398 (10.3%)	70 (4.5%)
Anxiety	275 (7.1%)	51 (3.3%)
PTSD	21 (0.5%)	7 (0.5%)
Bipolar	82 (2.1%)	28 (1.8%)
Schizophrenia	20 (0.5%)	10 (0.6%)
Substance use	421 (10.9%)	139 (9.0%)
Alcohol use disorder/poisoning	292 (7.6%)	101 (6.5%)
Drug use disorder/poisoning	208 (5.4%)	68 (4.4%)
Opioid use disorder/poisoning	70 (1.8%)	20 (1.3%)
Sedative/Hypnotic/Anxiolytic use disorder/poisoning	55 (1.4%)	14 (0.9%)
Cannabis use disorder	51 (1.3%)	16 (1.0%)
Stimulant use disorder/poisoning	36 (0.9%)	21 (1.4%)
Physical illness		
* Pain*	381 (9.9%)	109 (7.0%)
Rheumatoid Arthritis/Osteoarthritis	205 (5.3%)	63 (4.1%)
Migraine/Chronic Headache	47 (1.2%)	9 (0.6%)
Fibromyalgia/Chronic Pain/Fatigue	213 (5.5%)	52 (3.3%)
* Chronic disease*	971 (25.1%)	338 (21.8%)
Acute MI	51 (1.3%)	30 (1.9%)
Heart failure	150 (3.9%)	59 (3.8%)
Hypertension	785 (20.3%)	263 (16.9%)
Stroke/Transient Ischemic Attack	57 (1.5%)	29 (1.9%)
Asthma	104 (2.7%)	49 (3.2%)
COPD	204 (5.3%)	62 (4.0%)
Diabetes	258 (6.7%)	123 (7.9%)
TBI	9 (0.2%)	10 (0.6%)
Epilepsy	31 (0.8%)	26 (1.7%)
Cancer	135 (3.5%)	20 (1.3%)
Assault	35 (0.9%)	31 (2.0%)

*MVC* motor vehicle crash, *PSTD* post-traumatic stress disorder, *MI* myocardial infarction, *COPD* chronic obstructive pulmonary disorder, *TBI* traumatic brain injury

^a^Emergency department or hospital visit within 3 years prior to death

In multivariable models adjusting for demographic characteristics, death year, and county urbanicity, suicidal ideation/attempt (OR 4.92; 95% CI 3.27, 7.40), mental illness (OR 1.97; 95% CI 1.60, 2.43), alcohol use disorder (OR 1.29; 95% CI 1.01, 1.65), drug use disorder (OR 1.40; 95% CI 1.05, 1.88), and pain (OR 1.34; 95% CI 1.07, 1.69) were all associated with increased odds of firearm suicide (Table 2; Model 1). In the model adjusting for all health conditions simultaneously, only suicidal ideation/attempt and mental illness remained associated with increased odds of firearm suicide (Table 2; Model 2), and chronic diseases (OR 0.75; 95% CI 0.63, 0.90) and assault (OR 0.42; 95% CI 0.24, 0.75) were inversely associated with firearm suicide.

**Table 2:** Association between health diagnoses and firearm suicide among legal handgun purchasers (*N* = 5415)

	Model 1^a^			Model 2^b^		
	aOR	95% CI		aOR	95% CI	
Suicidal ideation/attempt	4.92	3.27	7.40	4.33	2.88	6.51
Mental illness	1.97	1.60	2.43	1.74	1.37	2.21
Alcohol use disorder/poisoning	1.29	1.01	1.65	1.06	0.80	1.40
Drug use disorder/poisoning	1.40	1.05	1.88	0.89	0.64	1.25
Pain	1.34	1.07	1.69	1.13	0.87	1.47
Chronic disease	0.99	0.85	1.15	0.75	0.63	0.90
Assault	0.54	0.32	0.92	0.42	0.24	0.75

*aOR* adjusted odds ratio, *CI* confidence intervals

^a^Estimates are from separate models, each adjusted for sex, age, death year, marital status, educational attainment, urbanicity

^b^Estimates are from a single model, adjusted for sex, age, death year, marital status, educational attainment, urbanicity

In multivariable models with disaggregated components for each clinical subcategory, depression; anxiety; opioid use disorder/poisoning; migraine, chronic headache; fibromyalgia, chronic pain, fatigue; and cancer were associated with increased odds of firearm suicide, whereas acute MI; heart failure; stroke; diabetes; TBI; and epilepsy were associated with lower odds of firearm suicide (Table 3; Model 1). Except for stimulant use disorder/poisoning, TBI, heart failure, hypertension, and stroke, results were consistent both when including each component in separate models (Table 3; Model 1) and when including them simultaneously in one model (Table 3; Model 2).

**Table 3:** Association between specific health diagnoses and firearm suicide among legal handgun purchasers (*N* = 5415)

	Model 1^a^			Model 2^b^		
	aOR	95% CI		aOR	95% CI	
Mental illness						
Depression	2.30	1.76	3.01	2.07	1.57	2.74
Anxiety	2.24	1.64	3.05	1.86	1.34	2.57
PTSD	1.58	0.66	3.78	1.07	0.44	2.65
Bipolar	1.26	0.79	1.98	0.80	0.49	1.29
Schizophrenia	0.71	0.33	1.55	0.52	0.22	1.25
Drug use						
Opioid use disorder/poisoning	1.74	1.01	2.97	1.68	0.96	2.97
Sedative/Hypnotic/Anxiolytic use disorder/poisoning	1.73	0.92	3.24	1.53	0.81	2.90
Cannabis use disorder	1.62	0.91	2.89	1.69	0.92	3.12
Stimulant use disorder/poisoning	0.80	0.45	1.42	0.55	0.30	1.03
Pain						
Rheumatoid Arthritis/Osteoarthritis	1.05	0.77	1.41	0.91	0.67	1.24
Migraine/Chronic Headache	2.47	1.18	5.17	2.20	1.04	4.66
Fibromyalgia/Chronic Pain/Fatigue	1.72	1.25	2.36	1.69	1.22	2.34
Chronic disease						
Acute MI	0.51	0.32	0.84	0.53	0.32	0.89
Heart failure	0.74	0.54	1.03	0.80	0.55	1.17
Hypertension	1.00	0.85	1.19	1.20	0.98	1.46
Stroke/Transient Ischemic Attack	0.62	0.38	1.01	0.68	0.41	1.12
Asthma	0.87	0.61	1.25	0.89	0.62	1.30
COPD	1.14	0.84	1.55	1.29	0.92	1.80
Diabetes	0.72	0.57	0.92	0.73	0.56	0.96
TBI	0.32	0.13	0.82	0.41	0.16	1.03
Epilepsy	0.42	0.24	0.73	0.46	0.26	0.81
Cancer	1.95	1.20	3.18	1.89	1.15	3.09

*aOR* adjusted odds ratio, *CI* confidence intervals

^a^Estimates are from separate models, each adjusted for sex, age, death year, marital status, educational attainment, urbanicity

^b^Estimates are from one model per each diagnosis category, each adjusted for sex, age, death year, marital status, educational attainment, urbanicity

**Fig. 1:**
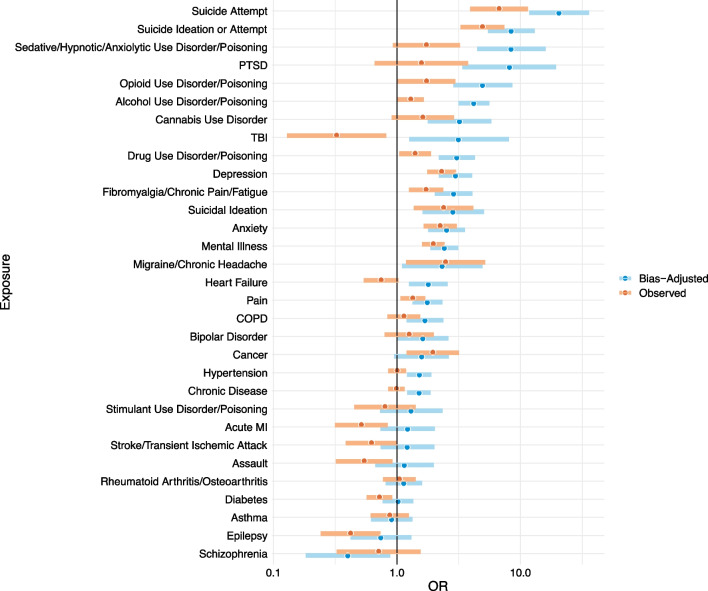
Observed and bias-adjusted odds ratios for firearm suicide using probabilistic quantitative selection bias analysis. *Note*: Odds ratios (ORs) are plotted on the log scale. Observed ORs are from separate models adjusted for sex, age, death year, marital status, educational attainment, urbanicity. The final bias-adjusted OR is adjusted for systematic selection bias and random error via the formula: $\text{exp}\left(\text{ln}({\widehat{\text{OR}}}_{\text{adj}})+N\left(\text{0,1}\right)\times \text{SE}(\text{ln}({\widehat{\text{OR}}}_{\text{observed}})\right)$ where ${\widehat{\text{OR}}}_{\text{adj}}= {\widehat{\text{OR}}}_{\text{observed}}\times {\text{OR}}_{\text{select}}$ where ${\widehat{\text{OR}}}_{\text{observed}}$ is the observed OR (adjusted for sex, age, death year, marital status, educational attainment, urbanicity) and ${\text{OR}}_{\text{select}}=\frac{{S}_{\text{case},0}{S}_{\text{control},1}}{{S}_{\text{case},1}{S}_{\text{control},0}}$ where S is a selection proportion and exposed and unexposed are indexed by 1 and 0, respectively. We assumed all cases were selected, thus ${\text{OR}}_{\text{select}}$ simplified to $\frac{{S}_{\text{control},1}}{{S}_{\text{control},0}}$. In the absence of known selection probabilities for the control group, we used the comparisons of exposure rates among MVC decedents and the general California population in eTable 1, Additional file 1 as a benchmark for ${\text{OR}}_{\text{select}}$. For example, the ${\text{OR}}_{\text{select}}$ for suicidal ideation/attempt was 8.00/4.67 = 1.71. We used a Monte Carlo simulation with 50,000 iterations, specifying a triangular distribution with mode equal to ${\text{OR}}_{\text{select}}$ and lower and upper limits ranging from 20% lower to 20% higher than the mode. The median of this distribution is the final bias-adjusted OR and the 2.5th and 97.5th percentiles constitute the 95% simulation interval

Results of our quantitative bias analysis suggest that observed associations were generally biased downward because the control group (MVC decedents) were disproportionately likely to have health diagnoses compared to the source population (Fig. 1). As such, after we adjusted for selection bias, most observed inverse associations became null (e.g., for diabetes, stroke, acute MI, and epilepsy), several observed null associations became positive (e.g., COPD, bipolar disorder), and almost all observed positive associations became stronger. For example, the bias-adjusted OR for suicidal ideation/attempt was 8.39 (95% simulation interval 5.46, 13.04), almost twice that of the observed OR.

## Discussion

In this case–control study of legal handgun purchasers in California, we found that hospital visits for suicidal ideation or attempt and mental illness in the three years prior to death were consistently associated with higher odds of firearm suicide. The association for suicidal ideation and attempt was especially strong; handgun purchasers who died by firearm suicide had over 4 times the odds of a prior visit for suicidal ideation/attempt compared with handgun purchasers who died in a motor vehicle crash. Depending on our modeling approach, alcohol and drug use disorders, pain diagnoses, and cancer were also positively associated with firearm suicide, whereas some chronic illnesses and assault were negatively associated with firearm suicide. Further disaggregated analyses showed that the results for mental illness were driven by diagnoses for depression and anxiety, and that opioid use disorder/poisoning was associated with higher odds of firearm suicide.

When we adjusted for selection bias using probabilistic quantitative bias analysis, these positive associations became even stronger, reflecting the over-representation of exposed controls in our sample. Differences between bias-adjusted and observed odds ratios tended to be greatest for exposures involving alcohol and drug use, which is unsurprising given the established association between motor vehicle crash risk and substance use (Li et al. 2013). We selected motor vehicle crash decedents as controls because this group provided a feasible trade-off between core principles of control selection: efficiency, comparable accuracy, study base, and deconfounding (Wacholder et al. 1992). We could not use the general living population of handgun purchasers as controls since we lacked data on hospital visits among non-deceased individuals. Therefore, using deceased controls was the only efficient strategy to obtain comparable data on handgun purchasers who did and did not die of firearm suicide. While motor vehicle crash decedents may not be representative of the general living population of handgun purchasers, this group can still be considered as arising from the same study base as members of the group could have been selected as a case had they died of firearm suicide (Murphy et al. 2017). Further, other potential control causes of death likely had very different distributions of potential confounders (e.g., socioeconomic status, health behaviors) compared with our cases, as this group tended to be much sicker generally than both firearm suicide decedents and motor vehicle crash decedents (eTable 2, Additional file 1).

A strength of our study is the use of probabilistic quantitative bias analysis to generate selection bias-adjusted estimates. Our bias analysis inherently (and explicitly) included uncertainty, since we did not know the burden of health conditions among the general living population of handgun purchasers. Instead, we benchmarked our estimates of selection bias against the general living population of California adults as a whole. To the extent that the exposure distribution of the general population of handgun purchasers was more similar to the general population of California adults than to handgun purchasers who died in motor vehicle crashes, our bias-adjusted estimates would be closer to the truth than observed estimates. That is, notwithstanding uncertainty in the selection bias parameters, our bias-adjusted estimates may provide a more realistic characterization of the associations between health conditions and suicide risk among handgun purchasers, while our observed (not bias-adjusted) odds ratios likely provide a conservative lower bound.

Our findings for mental illness and suicidal ideation and attempt are consistent with a large body of literature documenting strong associations between these diagnoses and risk of suicide death (Sivaraman et al. 2022; Ilgen et al. 2010; Olfson et al. 2021; Goldman-Mellor et al. 2019; Conner et al. 2019b). Prior research also suggests pain and substance use are associated with suicide (Sivaraman et al. 2022; Ilgen et al. 2010, 2013; Too et al. 2019; Conner et al. 2019b). We found that pain diagnoses and alcohol and drug use disorders generally were only associated with firearm suicide in models that did not simultaneously adjust for other health conditions. Other health conditions may be confounders of these associations; alternatively, they may be mediators, in which case adjustment could induce bias (Caves Sivaraman and Naumann 2020). Nevertheless, our minimally adjusted results indicate that hospital visits for alcohol and drug use disorders/poisoning and pain are indeed markers for elevated firearm suicide risk among handgun purchasers, even if the associations are explained in part by other factors. Further, when we disaggregated by substance, opioid use disorder/poisoning was consistently associated with higher odds of firearm suicide. This finding aligns with prior research and suggests that opioid use is uniquely related to suicide risk (Sivaraman et al. 2022; Bohnert et al. 2017; Bohnert and Ilgen 2019).

In contrast to existing research which has found chronic diseases to be positively associated with suicide (Hempstead et al. 2013; Goldman-Mellor et al. 2021; Ahmedani et al. 2017), we either found no relationship or a negative association, depending on how we grouped diagnoses, whether we adjusted for other health conditions, and whether we adjusted estimates for selection bias. Indeed, results of our quantitative bias analysis suggest that observed negative associations for chronic diseases reflected bias because our control group (those who died from motor vehicle crashes) was generally sicker than living controls. This is consistent with prior evidence, which has found that certain conditions, such as diabetes and myocardial infarction, are positively associated with motor vehicle crash risk (Cox et al. 2003; Dischinger et al. 2000; Sagberg 2006).

In addition, we found that approximately 45% of firearm suicide decedents had *any* hospital visit in the three years prior to death. This proportion is somewhat lower than that implied by prior studies among the general population or clinical samples (Ahmedani et al. 2014; Gairin et al. 2003; Cruz et al. 2011). This may be because firearm suicide decedents have less opportunity for healthcare intervention prior to attempt compared with those who use less lethal means (Caves Sivaraman and Naumann 2020). However, this large proportion nonetheless suggests that many firearm owners who died by firearm suicide intersected with the healthcare system relatively shortly before their death. Future research should investigate how best to leverage these moments of opportunity to reduce risk of suicide. Strategies might include lethal means safety counseling and reducing the burden of mental and physical health conditions through increased access to integrated health care and more foundational modifications to social determinants of health.

This study has several limitations. Due to limited data availability, data used in this study are several years old and might not generalize to more recent years. However, this analysis also provides an important historical account to contextualize more recent analyses and trends over time. More recent firearm purchasers differ demographically from previous firearm purchasers (e.g., more are women and people of color) (Miller et al. 2022; Wertz et al. 2018). Future research might examine how and if results differ to the extent that new firearm purchasers differ in their mental health, care-seeking (and receiving) behavior, and risk for suicide. Our findings might not generalize to non-handgun owners, those who purchased long guns (but not handguns), or those who purchased handguns illegally. In addition, health diagnoses may have measurement error insofar as clinicians may fail to record diagnoses or do so incorrectly. More likely is measurement error resulting from the fact that not all people with a given health condition end up in the ED or hospital to seek care. Nevertheless, the focus of our study was on potential opportunities for intervention during healthcare encounters, so we do not see this measurement error as a source of bias. Further, our goal in this study was to characterize rather than predict suicide risk; future research should seek to identify specific diagnostic profiles that are most predictive of risk among this population. Some diagnoses had small counts and estimates of their association were imprecise. Lastly, we estimated that our control group was more likely to suffer from health conditions than the general population of living handgun purchasers, which introduced a downward bias in our results. Our use of probabilistic QBA to quantify the degree of selection bias is a strength of the study.

## Conclusion

Results suggest that handgun purchasers with recent hospital visits for suicidal ideation or attempt are at substantially elevated risk of firearm suicide. Mental illness (namely depression and anxiety) and substance use disorders (namely opioid use disorder) were also positively associated with firearm suicide. These findings, and the large proportion of firearm suicide decedents with any prior healthcare encounter, indicate that healthcare encounters may be useful settings for identifying firearm owners at elevated risk of suicide.

## Supplementary Information

**Additional file 1:** Supplementary Material.

## Data Availability

The data that support the findings of this study are available from the California Department of Justice, California Department of Public Health, and Office of Statewide Planning Health and Development but restrictions apply to the availability of these data, which were used under license for the current study, and so are not publicly available.

## Abbreviations

DROS: Dealer’s Record of Sale
CA DOJ: California Department of Justice
OSHPD: Office of Statewide Health Planning and Development
ICD: International Classification of Diseases
ED: Emergency department
MVC: Motor vehicle crash
CM: Clinical modification
PTSD: Post-traumatic stress disorder
MI: Myocardial infarction
COPD: Chronic obstructive pulmonary disorder
TBI: Traumatic brain injury
QBA: Quantitative bias analysis

## References

[CR1] Ahmedani BK, Simon GE, Stewart C, Beck A, Waitzfelder BE, Rossom R (2014). Health care contacts in the year before suicide death. J Gen Intern Med.

[CR2] Ahmedani BK, Peterson EL, Hu Y, Rossom RC, Lynch F, Lu CY (2017). Major physical health conditions and risk of suicide. Am J Prev Med.

[CR3] Anglemyer A, Horvath T, Rutherford G (2014). The accessibility of firearms and risk for suicide and homicide victimization among household members: a systematic review and meta-analysis. Ann Intern Med.

[CR4] Banack HR, Hayes-Larson E, Mayeda ER (2022). Monte Carlo simulation approaches for quantitative bias analysis: a tutorial. Epidemiol Rev.

[CR5] Boggs JM, Beck A, Hubley S, Peterson EL, Hu Y, Williams LK (2018). Critical suicide risk factors: an examination of mental health, medical and demographic factors for patients who die by firearm compared to other means. Psychiatr Serv.

[CR6] Bohnert ASB, Ilgen MA (2019). Understanding links among opioid use, overdose, and suicide. N Engl J Med.

[CR7] Bohnert KM, Ilgen MA, Louzon S, McCarthy JF, Katz IR (2017). Substance use disorders and the risk of suicide mortality among men and women in the US Veterans Health Administration. Addiction.

[CR8] Caves Sivaraman JJ, Naumann RB (2020). Estimating the association between mental health disorders and suicide: a review of common sources of bias and challenges and opportunities for US-based research. Curr Epidemiol Rep.

[CR9] Conner A, Azrael D, Miller M (2019). Suicide case-fatality rates in the United States, 2007 to 2014: a nationwide population-based study. Ann Intern Med.

[CR10] Conner KR, Bridge JA, Davidson DJ, Pilcher C, Brent DA (2019). Metaanalysis of mood and substance use disorders in proximal risk for suicide deaths. Suicide Life Threat Behav.

[CR11] Cox DJ, Penberthy JK, Zrebiec J, Weinger K, Aikens JE, Frier B (2003). Diabetes and driving mishaps: frequency and correlations from a multinational survey. Diabetes Care.

[CR12] Cruz DD, Pearson A, Saini P, Miles C, While D, Swinson N (2011). Emergency department contact prior to suicide in mental health patients. Emerg Med J.

[CR13] Cunningham RM, Walton MA, Carter PM (2018). The major causes of death in children and adolescents in the United States. N Engl J Med.

[CR14] Dischinger PC, Ho SM, Kufera JA (2000). Medical conditions and car crashes. Annu Proc Assoc Adv Automot Med.

[CR15] Douthit N, Kiv S, Dwolatzky T, Biswas S (2015). Exposing some important barriers to health care access in the rural USA. Public Health.

[CR16] Gairin I, House A, Owens D (2003). Attendance at the accident and emergency department in the year before suicide: retrospective study. Br J Psychiatry J Ment Sci.

[CR17] Goldman-Mellor S, Olfson M, Lidon-Moyano C, Schoenbaum M (2019). Association of suicide and other mortality with emergency department presentation. JAMA Netw Open.

[CR18] Goldman-Mellor S, Hall C, Cerdá M, Bhat H (2021). Firearm suicide mortality among emergency department patients with physical health problems. Ann Epidemiol.

[CR19] Hempstead K, Nguyen T, David-Rus R, Jacquemin B (2013). Health problems and male firearm suicide. Suicide Life Threat Behav.

[CR20] Ilgen MA, Bohnert ASB, Ignacio RV, McCarthy JF, Valenstein MM, Kim HM (2010). Psychiatric diagnoses and risk of suicide in veterans. Arch Gen Psychiatry.

[CR21] Ilgen MA, Kleinberg F, Ignacio RV, Bohnert ASB, Valenstein M, McCarthy JF (2013). Noncancer pain conditions and risk of suicide. JAMA Psychiat.

[CR22] Kagawa RMC, Stewart S, Wright MA, Shev AB, Pear VA, McCort CD (2020). Association of prior convictions for driving under the influence with risk of subsequent arrest for violent crimes among handgun purchasers. JAMA Intern Med.

[CR23] Kellermann AL, Rivara FP, Somes G, Reay DT, Francisco J, Banton JG (1992). Suicide in the home in relation to gun ownership. N Engl J Med.

[CR24] Lash TL, Fox MP, Fink AK (2009). Applying quantitative bias analysis to epidemiologic data.

[CR25] Li G, Brady JE, Chen Q (2013). Drug use and fatal motor vehicle crashes: a case–control study. Accid Anal Prev.

[CR26] Miller M, Zhang W, Azrael D (2022). Firearm purchasing during the COVID-19 pandemic: results from the 2021 national firearms survey. Ann Intern Med.

[CR27] Murphy B, Ibrahim JE, Bugeja L, Pilgrim J, Cicuttini F (2017). The use of deceased controls in epidemiologic research: a systematic review. Am J Epidemiol.

[CR28] Olfson M, Gao YN, Xie M, Wiesel Cullen S, Marcus SC (2021). Suicide risk among adults with mental health emergency department visits with and without suicidal symptoms. J Clin Psychiatry.

[CR29] Oliva EM, Bowe T, Tavakoli S, Martins S, Lewis ET, Paik M (2017). Development and applications of the Veterans Health Administration’s Stratification Tool for Opioid Risk Mitigation (STORM) to improve opioid safety and prevent overdose and suicide. Psychol Serv.

[CR30] Pallin R, Barnhorst A (2021). Clinical strategies for reducing firearm suicide. Inj Epidemiol.

[CR31] Pallin R, Spitzer SA, Ranney ML, Betz ME, Wintemute GJ (2019). Preventing firearm-related death and injury. Ann Intern Med.

[CR32] Sagberg F (2006). Driver health and crash involvement: a case–control study. Accid Anal Prev.

[CR33] Schleimer JP, Wright MA, Shev AB, McCort CD, Asif-Sattar R, Sohl S (2021). Alcohol and drug offenses and suicide risk among men who purchased a handgun in California: a cohort study. Prev Med.

[CR34] Sivaraman JJC, Greene SB, Naumann RB, Proescholdbell S, Ranapurwala SI, Marshall SW (2022). Association between medical diagnoses and suicide in a medicaid beneficiary population, North Carolina 2014–2017. Epidemiology.

[CR35] Studdert DM, Zhang Y, Swanson SA, Prince L, Rodden JA, Holsinger EE (2020). Handgun ownership and suicide in California. N Engl J Med.

[CR36] Too LS, Spittal MJ, Bugeja L, Reifels L, Butterworth P, Pirkis J (2019). The association between mental disorders and suicide: a systematic review and meta-analysis of record linkage studies. J Affect Disord.

[CR37] Tordoff D, Andrasik M, Hajat A (2019). Misclassification of sex assigned at birth in the behavioral risk factor surveillance system and transgender reproductive health: a quantitative bias analysis. Epidemiology.

[CR38] Wacholder S, McLaughlin JK, Silverman DT, Mandel JS (1992). Selection of controls in case–control studies I. Principles. Am J Epidemiol.

[CR39] Wertz J, Azrael D, Hemenway D, Sorenson S, Miller M (2018). Differences between new and long-standing US gun owners: results from a national survey. Am J Public Health.

[CR40] Wintemute GJ, Parham CA, Beaumont JJ, Wright M, Drake C (1999). Mortality among recent purchasers of handguns. N Engl J Med.

[CR41] CDC WISQARS. Fatal injury and violence data. [cited 2022 Jun 29]. Available from: https://www.cdc.gov/injury/wisqars/fatal.html

[CR42] CDC’s Drug Overdose Surveillance and Epidemiology (DOSE) System | Drug Overdose | CDC Injury Center. 2022 [cited 2022 Aug 17]. Available from: https://www.cdc.gov/drugoverdose/nonfatal/case.html

[CR43] Centers for Medicare and Medicaid Services. Condition Categories Data Warehouse. Chronic Conditions Data Warehouse. [cited 2022 Aug 17]. Available from: https://www2.ccwdata.org/condition-categories

[CR44] Fingar KR, Skinner H, Johann J, Coenen N, Freeman WJ, Heslin KC. Geographic variation in substance-related inpatient stays across states and counties in the United States, 2013–2015; Table 3, Definition of substance use. In: Healthcare Cost and Utilization Project (HCUP) Statistical Briefs. Rockville (MD): Agency for Healthcare Research and Quality (US); 2018 [cited 2022 Aug 24]. Available from: https://www.ncbi.nlm.nih.gov/books/NBK537456/table/sb245.tab3/

[CR45] Garnett MF, Curtin S, Stone D. Suicide mortality in the United States, 2000–2020. 2022 p. 8. Available from: https://www.cdc.gov/nchs/data/databriefs/db433.pdf 35312475

[CR46] Heslin KC, Elixhauser A, Steiner CA. Table 4, ICD-9-CM diagnosis codes defining substance use disorders. Agency for Healthcare Research and Quality (US); 2015 [cited 2022 Aug 17]. Available from: https://www.ncbi.nlm.nih.gov/books/NBK310986/table/sb191.t4/

[CR47] Mitchell T. The demographics of gun ownership. Pew Research Center’s Social & Demographic Trends Project. 2017 [cited 2023 May 18]. Available from: https://www.pewresearch.org/social-trends/2017/06/22/the-demographics-of-gun-ownership/

[CR48] National Center for Injury Prevention and Control. Prescription Drug Overdose Data & Statistics Guide To ICD-9-CM and ICD-10 Codes Related To Poisoning and Pain. Atlanta, GA: Centers for Disease Control and Prevention; 2013 [cited 2021 Oct 1]. Available from: https://www.google.com/url?sa=t&rct=j&q=&esrc=s&source=web&cd=&cad=rja&uact=8&ved=2ahUKEwjwibfGuc_5AhXgHjQIHSAqCvYQFnoECAcQAQ&url=https%3A%2F%2Fstacks.cdc.gov%2Fview%2Fcdc%2F59394&usg=AOvVaw03JqqGY2N-sCTwGR_2rHeE

[CR49] Rothman KJ, Greenland S, Lash TL. Case–control studies. In: Modern epidemiology. 3rd ed. Philadelphia: Wolters Kluwer Health/Lippincott Williams & Wilkins; 2008.

[CR50] United States Department of Agriculture. Rural–Urban Continuum Codes. [cited 2021 Sep 14]. Available from: https://www.ers.usda.gov/data-products/rural-urban-continuum-codes/

