# Supplementary material for: Association of medical conditions and firearm suicide among legal handgun purchasers in California: a case–control study
Source: Inj Epidemiol. 2023 Jun 16;10:26. doi: 10.1186/s40621-023-00437-6 (PMC10276506; doi:10.1186/s40621-023-00437-6)
Supplement: Supplementary file 1 — Additional file 1: Supplementary Material. [file 40621_2023_437_MOESM1_ESM.docx]

**Additional File 1**

Association of Medical Conditions and Firearm Suicide Among Legal Handgun Purchasers in California: A Case-Control Study

Julia P. Schleimer,^1,2^ Rose M.C. Kagawa,^1,2^ Hannah S. Laqueur^1,2^

^1^Department of Emergency Medicine, Violence Prevention Research Program, University of California, Davis School of Medicine, Sacramento, CA, US

^2^California Firearm Violence Research Center, Sacramento, CA, USA

eTable 1. International Classification of Diseases (ICD)-9-Clincal Modification (CM) Codes

|  | ICD-9-CM codes | Paired with E-codes* |
| --- | --- | --- |
| **Suicidal ideation/attempt** |  |  |
| Suicidal ideation | V6284 |  |
| Suicide attempt | E9500, E9501, E9502, E9503, E9504, E9505, E9506, E9507, E9508, E9509, E9510, E9511, E9518, E9520, E9521, E9528, E9529, E9530, E9531, E9538, E9539, E954 , E9550, E9551, E9552, E9553, E9554, E9555, E9556, E9557, E9559, E956 , E9570, E9571, E9572, E9579, E9580, E9581, E9582, E9583, E9584, E9585, E9586, E9587, E9588, E9589, E959 |  |
| **Mental illness** |  |  |
| Depression | 29620, 29621, 29622, 29623, 29624, 29625, 29626, 29630, 29631, 29632, 29633, 29634, 29635, 29636, 2980, 3004, 311, 3091 |  |
| Anxiety | 29384, 30000, 30001, 30002, 30009, 30020, 30021, 30022, 30023, 30029, 3003, 3080, 3081, 3082, 3084, 3089, 30921, 3130, 31321, 30010, 3005, 30089, 3009, 3131, 31322, 3133, 31382, 31383 |  |
| PTSD | 30981 |  |
| Bipolar | 29600, 29601, 29602, 29603, 29604, 29605, 29606, 29610, 29611, 29612, 29613, 29614, 29615, 29616, 29640, 29641, 29642, 29643, 29644, 29645, 29646, 29650, 29651, 29652, 29653, 29654, 29655, 29656, 29660, 29661, 29662, 29663, 29664, 29665, 29666, 2967, 29680, 29681, 29689, 29682, 29690, 29699 |  |
| Schizophrenia | 29500, 29501, 29502, 29503, 29504, 29505, 29510, 29511, 29512, 29513, 29514, 29515, 29520, 29521, 29522, 29523, 29524, 29525, 29530, 29531, 29532, 29533, 29534, 29535, 29540, 29541, 29542, 29543, 29544, 29545, 29550, 29551, 29552, 29553, 29554, 29555, 29560, 29561, 29562, 29563, 29564, 29565, 29570, 29571, 29572, 29573, 29574, 29575, 29580, 29581, 29582, 29583, 29584, 29585, 29590, 29591, 29592, 29593, 29594, 29595 |  |
| **Substance use** |  |  |
| Alcohol use disorder | 2910, 2911, 2912, 2913, 2914, 2915, 2918, 2919, 3575, 4255, 5710, 5711, 5712, 5713, 9800, 29181, 29182, 29189, 30300, 30301, 30302, 30390, 30391, 30392, 30500, 30501, 30502, 53530, 53531, 76071, E8600 |  |
| Drug use disorder | 3041, 2929, 304, 2920, 2922, 3040, 3042, 3043, 3044, 3045, 3046, 3047, 3048, 3049, 3052, 3053, 3054, 3055, 3056, 3057, 3058, 3059, 6483, 6555, 29211, 29212, 29281, 29282, 29283, 29284, 29285, 29289, 30400, 30401, 30402, 30410, 30411, 30412, 30420, 30421, 30422, 30430, 30431, 30432, 30440, 30441, 30442, 30450, 30451, 30452, 30460, 30461, 30462, 30470, 30471, 30472, 30480, 30481, 30482, 30490, 30491, 30492, 30520, 30521, 30522, 30530, 30531, 30532, 30540, 30541, 30542, 30550, 30551, 30552, 30560, 30561, 30562, 30570, 30571, 30572, 30580, 30581, 30582, 30590, 30591, 30592, 64830, 64831, 64832, 64833, 64834, 65550, 65551, 65553, E8500, E8501, E8502, E851 , E8520, E8521, E8522, E8523, E8524, E8525, E8528, E8529, E8530, E8531, E8532, E8538, E8539, E8541, E8542, E8543, E8548, E8588, E8589 | 9650, 9670, 9671, 9672, 9673, 9674, 9675, 9676, 9678, 9679, 9680, 9685, 9691, 9692, 9693, 9694, 9695, 9696, 9698, 9699, 9700, 9708, 9709, 96500, 96501, 96502, 96509, 96970, 96971, 96972, 96973, 96979, 97081, 97089 |
| Opioid use disorder/ poisoning | 304, 3040, 3047, 3055, 30400, 30401, 30402, 30470, 30471, 30472, 30550, 30551, 30552, E8500, E8501, E8502 | 9650, 96500, 96501, 96502, 96509 |
| Sedative/ Hypnotic/ Anxiolytic use disorder/ poisoning | 3041, 3054, 30410, 30411, 30412, 30540, 30541, 30542, E851, E8520, E8521 , E8522 , E8523 , E8524 , E8525 , E8528 , E8529 , E8530 , E8531, E8532, E8538, E8539 | 9670, 9671, 9672, 9673, 9674, 9675, 9676, 9678, 9679, 9680, 9691, 9692, 9693, 9694, 9695 |
| Cannabis use disorder | 3043, 3052, 30430, 30431, 30432, 30520, 30521, 30522 |  |
| Stimulant use disorder/ poisoning | 3042, 3044, 3056, 3057, 30420, 30421, 30422, 30440, 30441, 30442, 30560, 30561, 30562, 30570, 30571, 30572, E8542, E8543 | 9685, 9700, 9708, 9709, 96970, 96971, 96972, 96973, 96979, 97081, 97089 |
| **Physical illness** |  |  |
| *Pain* |  |  |
| Rheumatoid Arthritis/ Osteoarthritis | 7140, 7141, 7142, 71430, 71431, 71432, 71433, 71500, 71504, 71509, 71510, 71511, 71512, 71513, 71514, 71515, 71516, 71517, 71518, 71520, 71521, 71522, 71523, 71524, 71525, 71526, 71527, 71528, 71530, 71531, 71532, 71533, 71534, 71535, 71536, 71537, 71538, 71580, 71589, 71590, 71591, 71592, 71593, 71594, 71595, 71596, 71597, 71598, 7200, 7210, 7211, 7212, 7213, 72190, 72191 |  |
| Migraine/ Chronic Headache | 339, 3390, 33900, 33901, 33902, 33903, 33904, 33905, 33909, 3391, 33910, 33911, 33912, 3392, 33920, 33921, 33922, 3393, 3394, 33941, 33942, 33943, 33944, 3398, 33981, 33982, 33983, 33984, 33985, 33989, 346, 3460, 34600, 34601, 34602, 34603, 3461, 34610, 34611, 34612, 34613, 3462, 34620, 34621, 34622, 34623, 3463, 34630, 34631, 34632, 34633, 3464, 34640, 34641, 34642, 34643, 3465, 34650, 34651, 34652, 34653, 3466, 34660, 34661, 34662, 34663, 3467, 34670, 34671, 34672, 34673, 3468, 34680, 34681, 34682, 34683, 3469, 34690, 34691, 34692, 34693 |  |
| Fibromyalgia/ Chronic Pain/ Fatigue | 3382, 33821, 33822, 33828, 33829, 3383, 3384, 7807, 78071, 7291, 7292 |  |
| *Chronic disease* |  |  |
| Acute MI | 41001, 41011, 41021, 41031, 41041, 41051, 41061, 41071, 41081, 41091 |  |
| Heart failure | 39891, 40201, 40211, 40291, 40401, 40403, 40411, 40413, 40491, 40493, 4280, 4281, 42820, 42821, 42822, 42823, 42830, 42831, 42832, 42833, 42840, 42841, 42842, 42843, 4289 |  |
| Hypertension | 36211, 4010, 4011, 4019, 40200, 40201, 40210, 40211, 40290, 40291, 40300, 40301, 40310, 40311, 40390, 40391, 40400, 40401, 40402, 40403, 40410, 40411, 40412, 40413, 40490, 40491, 40492, 40493, 40501, 40509, 40511, 40519, 40591, 40599, 4372 |  |
| Stroke/ Transient Ischemic Attack | 430, 431, 43301, 43311, 43321, 43331, 43381, 43391, 43400, 43401, 43410, 43411, 43490, 43491, 4350, 4351, 4353, 4358, 4359, 436, 99702 |  |
| Asthma | 49300, 49301, 49302, 49310, 49311, 49312, 49320, 49321, 49322, 49381, 49382, 49390, 49391, 49392 |  |
| COPD | 490, 4910, 4911, 49120, 49121, 49122, 4918, 4919, 4920, 4928, 4940, 4941, 496 |  |
| Diabetes | 24900, 24901, 24910, 24911, 24920, 24921, 24930, 24931, 24940, 24941, 24950, 24951, 24960, 24961, 24970, 24971, 24980, 24981, 24990, 24991, 25000, 25001, 25002, 25003, 25010, 25011, 25012, 25013, 25020, 25021, 25022, 25023, 25030, 25031, 25032, 25033, 25040, 25041, 25042, 25043, 25050, 25051, 25052, 25053, 25060, 25061, 25062, 25063, 25070, 25071, 25072, 25073, 25080, 25081, 25082, 25083, 25090, 25091, 25092, 25093, 3572, 36201, 36202, 36203, 36204, 36205, 36206, 36641 |  |
| TBI | 310, 3100, 3101, 3102, 3108, 31081, 31089, 907, 9070, 9071 |  |
| Epilepsy | 345, 3450, 34500, 34501, 3451, 34510, 34511, 3452, 3453, 3454, 34540, 34541, 3455, 34550, 34551, 3456, 34560, 34561, 3457, 34570, 34571, 3458, 34580, 34581, 3459, 34590, 34591 |  |
| Cancer | 1740, 1741, 1742, 1743, 1744, 1745, 1746, 1748, 1749, 1750, 1759, 2330, V103, 1530, 1531, 1532, 1533, 1534, 1535, 1536, 1537, 1538, 1539, 1540, 1541, 2303, 2304, V1005, V1006, 1820, 2332, V1042, 1622, 1623, 1624, 1625, 1628, 1629, 2312, V1011, 185, 2334, V1046 |  |
| **Assault** | E961, E9620, E9621, E9622, E9629, E963, E964, E9655, E9656, E9657, E9658, E9659, E966, E9680, E9681, E9682, E9683, E9684, E9685, E9686, E9687, E9688, E9689, E9600 |  |

*Poisoning diagnosis codes with any E-code for self-harm (E950 E951 E952); assault/ homicide (E962); legal intervention or operation of war (E972, E997.1, E997.2); or undetermined intent (E980, E981, E982) were excluded.

PSTD = post-traumatic stress disorder; MI = myocardial infarction; COPD = chronic obstructive pulmonary disorder; TBI = traumatic brain injury

eTable 2. Comparison of Rates of Emergency Department and Hospital Visits for Health Conditions Among Deceased Handgun Purchasers by Cause of Death and the General California Population

|  | Legal handgun purchasers* | | |  |
| --- | --- | --- | --- | --- |
|  | Firearm suicide decedents | MVC decedents | All other causes of death | General population^†^ |
| **Suicidal ideation/attempt** | 30.51 | 8.00 | 8.02 | 4.67 |
| Suicidal ideation | 9.60 | 4.00 | 5.65 | 3.36 |
| Suicide attempt | 20.91 | 4.00 | 2.37 | 1.31 |
| **Mental illness** | 114.60 | 65.91 | 216.57 | 53.52 |
| Depression | 58.88 | 27.37 | 123.62 | 21.10 |
| Anxiety | 35.66 | 20.43 | 59.86 | 18.09 |
| PTSD | 2.74 | 5.26 | 3.96 | 1.02 |
| Bipolar | 13.89 | 9.48 | 18.01 | 7.33 |
| Schizophrenia | 3.43 | 3.37 | 11.12 | 5.99 |
| **Substance use** | 83.40 | 79.39 | 149.45 | 28.72 |
| Alcohol use disorder | 53.31 | 51.17 | 107.09 | 15.78 |
| Drug use disorder/poisoning | 30.08 | 28.22 | 42.36 | 12.95 |
| Opioid use disorder/poisoning | 9.86 | 9.05 | 14.16 | 3.17 |
| Sedative/Hypnotic/Anxiolytic use disorder/poisoning | 5.91 | 3.58 | 3.47 | 0.74 |
| Cannabis use disorder | 5.49 | 6.32 | 5.78 | 3.18 |
| Stimulant use disorder/poisoning | 4.20 | 6.74 | 9.05 | 4.13 |
| **Physical illness** |  |  |  |  |
| *Pain* | 70.80 | 54.54 | 204.86 | 41.50 |
| Rheumatoid Arthritis/Osteoarthritis | 27.08 | 19.37 | 105.04 | 17.81 |
| Migraine/Chronic Headache | 8.06 | 5.69 | 9.14 | 6.05 |
| Fibromyalgia/Chronic Pain/Fatigue | 35.66 | 29.48 | 90.68 | 17.63 |
| *Chronic disease* | 385.19 | 380.71 | 2168.58 | 249.27 |
| Acute MI | 5.91 | 7.37 | 39.01 | 3.11 |
| Heart failure | 31.80 | 46.54 | 307.61 | 19.26 |
| Hypertension | 176.22 | 161.93 | 753.31 | 106.72 |
| Stroke/Transient Ischemic Attack | 5.83 | 8.21 | 39.98 | 4.21 |
| Asthma | 15.00 | 21.27 | 65.79 | 20.48 |
| COPD | 44.31 | 31.38 | 276.25 | 21.16 |
| Diabetes | 70.03 | 76.23 | 460.97 | 53.77 |
| TBI | 0.94 | 5.05 | 2.31 | 0.52 |
| Epilepsy | 4.63 | 12.00 | 37.40 | 6.83 |
| Cancer | 30.51 | 10.74 | 185.96 | 13.21 |
| **Assault** | 3.43 | 7.16 | 2.62 | 3.38 |

*Rate per 1,000 person-years among legal handgun purchasers in California who died between 2008-2013 (aged 21 and older at the time of death)

^†^Rate per 1,000 person-years among all Californians aged 21 and older, 2009-2013 (data for prior years were not available)

PSTD = post-traumatic stress disorder; MI = myocardial infarction; COPD = chronic obstructive pulmonary disorder; TBI = traumatic brain injury

**Example code for probablistic Quantitative Bias Analysis (QBA) for selection bias:**

## function for bias-adjusted OR

select_function <- function(se, or, min, max, mode){

df <- setNames(data.frame(matrix(ncol = 7, nrow = 50000)), c("ORobserved", "se","norm","se*norm", "ORselect","ORadj","ORadj_br"))

df$ORobserved <- or # observed OR (adjusted for basics)

df$se <- se # robust SE from model that generated observed OR

for (i in 1:50000) {

df[i,3] <- rnorm(1, mean=0, sd=1) # sample from standard normal (random0,1)

df[i,4] <- se*df[i,3] # se * norm

}

for (i in 1:50000) {

df[i,5] <- EnvStats::rtri(1, min, max, mode) # sample from trangular dist for OR select

}

for (i in 1:50000) {

df[i,6] <- log(df[i,1]*df[i,5]) # ln(ORadj) = ln(ORobserved * ORselect)

}

for (i in 1:50000) {

df[i,7] <- exp(df[i,6]+df[i,4]) # ORadj_br = exp(ln(ORadj) + (SE(ln(ORobserved)) * norm))

}

return(df)

}

## generate bias-adjusted estimate for prior suicidal ideation or attempt

model <- regress("odds", firearmSuicide ~ GENDER + age + death_year + marital2 + education2+rural_urban+suicide_risk, data = dat2) # regress uses robust SEs by default

se <- coef(model)[16,3] # SE for ln OR

or <- coef(model)[16,4] # ln OR

mode <- 1.71306210 # exposure rate among MVC decedents vs. the general CA population for this exposure

min <- mode - (0.2*mode)

max <- mode + (0.2*mode)

t <- select_function(se, or, min, max, mode)

quantile(t$ORadj_br, probs = c(.0275, .50, .975))
